# Novel Molecular Leads for the Prevention of Damage and the Promotion of Repair in Neuroimmunological Disease

**DOI:** 10.3389/fimmu.2019.01657

**Published:** 2019-07-19

**Authors:** Mahshad Kolahdouzan, Naomi C. Futhey, Nicholas W. Kieran, Luke M. Healy

**Affiliations:** Neuroimmunology Unit, Department of Neurology and Neurosurgery, Montreal Neurological Institute, McGill University, Montréal, QC, Canada

**Keywords:** neuroimmunology, inflammation, repair, multiple sclerosis, therapeutics, remyelination

## Abstract

Neuroinflammation is a prominent pathological feature of all neuroimmunological diseases, including, but not limited to, multiple sclerosis (MS), myasthenia gravis, neuromyelitis optica, and Guillain–Barré syndrome. All currently-approved therapies for the treatment of these diseases focus on controlling or modulating the immune (innate and adaptive) responses to limit demyelination and neuronal damage. The primary purpose of this review is to detail the pre-clinical data and proposed mechanism of action of novel drugs currently in clinical trial, with a focus on novel compounds that promote repair and regeneration in the central nervous system (CNS). As the most recent advances have been made in the field of MS research, this review will focus primarily on this disease and its animal models. However, these compounds are likely to be effective for a range of indications with a neuroinflammatory component. Traditionally, MS was thought to proceed through two distinct phases. The first, predominantly inflammatory stage, is characterized by acute episodes of clinical relapse, followed by periods of partial or total recovery with an apparent absence of overall disease progression. In the vast majority of patients, this relapsing-remitting disease subsequently progresses into a second more chronic, neurodegenerative phase, which is characterized by oligodendrocyte damage and axonal destruction leading to brain atrophy and an accumulation of disability. Recent work has shown that rather than occurring independently, both the inflammatory and degenerative phases may run concurrently. This, combined with evidence that early therapeutic intervention slows accumulation of disability and delays progression, highlights the need for novel therapeutic approaches that promote repair and regeneration early in the disease trajectory. Such compounds may be used as monotherapies or in conjunction with classical anti-inflammatory therapies. This review will highlight novel therapies currently in clinical trial, and likely to appear in clinical practice in the near future, focusing on compounds that target the immune system and/or enhance endogenous repair mechanisms in the CNS.

## Introduction

Neuroimmunology is the multidisciplinary study of the interaction between the immune, central, and peripheral nervous systems. One can consider four classical immunological diseases of the nervous system: multiple sclerosis (MS), Guillain-Barré syndrome, myasthenia gravis, and neuromyelitis optica (NMO). Neuroinflammation is a term often closely and correctly associated with these diseases and has traditionally been used to describe the infiltration of blood-derived leukocytes, the dysregulation of the blood-brain-barrier, and a considerable, and sustained glial activation. While the sequence of these events is still in question, the net accumulation of peripheral immune cells in the central nervous system (CNS) is not. “Neuroinflammation” is a common thread that binds these diseases. However, in the past decade this moniker has been appropriated to describe changes (specifically in resident glia) that fall within the confines of a normal glial response. These include changes during the aging process, response to social stress, and intrinsic (obesity), and extrinsic (air pollution) stressors (1, 2). The esoteric nature of neuroimmunology and the more loose application of the term “neuroinflammation” has brought more and more diseases under the neuroimmunological disease umbrella. Any change to brain homeostasis induces a microglial and astrocytic response often predicated on a release of immune-active molecules. It could therefore be argued that all diseases of the brain and spinal cord are, by virtue of the fact that they induce such glia-mediated changes, neuroimmunological in nature. The advent of “omics” level-interrogation of human disease tissue in conjunction with large, highly reproducible genome-wide association studies (GWAS) have positioned immune cells and their products at the center of discussions on the pathological causes and drivers of neurological disease. These include neurodegenerative diseases (Alzheimer's, Parkinson's, Huntington's and amyotrophic lateral sclerosis), neurodevelopmental disorders such as autism spectrum disorders, and mental health illnesses such as schizophrenia (3).

MS has long been the prototypical inflammatory disease of the CNS Since MS is a neuroimmunological disease with a distinct degenerative component, the complexity and evolving nature of MS pathogenesis provides an intriguing nexus point between inflammatory and degenerative cellular and molecular processes. While great inroads have been made in treating the inflammatory aspects of early MS, therapeutic options for patients with progressive disease remain sorely lacking. The extent to which neuroinflammation and neurodegeneration occur independently, in parallel, or sequentially is still an area of intense study. It is clear that the prolonged multiphasic nature of MS-associated neuroinflammation invariably leads to neurodegeneration and brain volume loss. Interestingly, this is not the case in some other neuroimmunological disease such as NMO, which is caused by a classic autoimmune response directed against the water channel, aquaporin-4, expressed on astrocytes (4). In NMO, the majority of patients do not experience a progressive phase of the disease. The MS field is currently shifting its attention to identifying novel, highly directed anti-inflammatory therapies and growth/repair-promoting compounds to tackle both inflammatory and progressive forms of disease. New and exciting targets are being identified with the goal of treating the inflammatory component of the disease in a more specific manner, thereby mitigating against unforeseen and potentially negative consequences of an untargeted immunosuppression. At the same time, novel methods to promote repair and remyelination are being explored, with great successes being reported in pre-clinical and early clinical trial studies.

MS is a multifocal, demyelinating, inflammatory disease of the CNS characterized by oligodendrocyte loss and progressive neurodegeneration, as shown in Figure 2A. The development of MS is thought to be at least in part caused by a loss of homeostatic immunological control measures that normally prevent the progression of benign autoimmune responses to pathogenic autoimmunity (5). This loss of homeostatic control is itself thought to be a result of a combination of genetic predisposition, hormonal changes and environmental triggers. Clinical symptoms vary based on the site of neurologic lesions and often correlate with invasion of inflammatory cells across the blood-brain barrier with resulting demyelination and edema (6). The McDonald criteria for MS diagnosis requires two or more clinical episodes with two or more demyelinating lesions on MRI, appearing in separate loci over time. Patients with clinically isolated syndrome (optic neuritis, brain-stem dysfunction, or incomplete transverse myelitis) have a greater chance of conversion to relapsing-remitting MS (RRMS) depending upon the lesion load. RRMS typically manifests in the second or third decade of life (7). Seventy percent of these RRMS patients progress to secondary progressive MS (SPMS) which is characterized by irreversible, progressive accumulation of disability (7). Ten to fifteen percent of MS patients are diagnosed with a primary progressive form (PPMS); these patients experience progressive disease after initial symptoms without relapses, with a similar incidence among men and women (6, 7).

**Figure 1:**
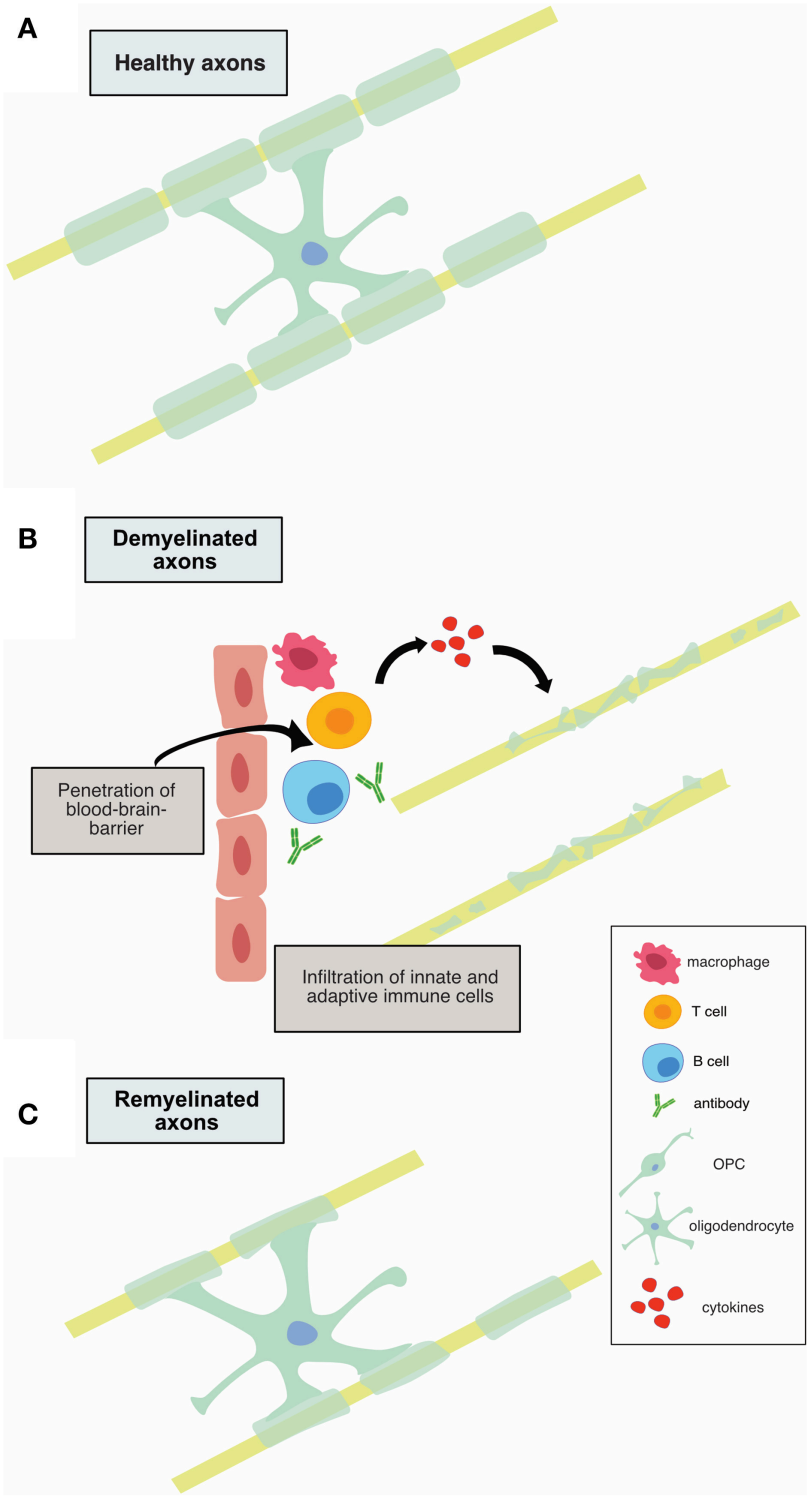
Illustration of demyelination and remyelination in MS. **(A)** shows a healthy, myelinated axon. In **(B)**, infiltration of innate and adaptive immune cells is shown, along with subsequent demyelination of axons. **(C)** depicts the remyelination process that occurs in relapsing-remitting MS, in which the myelin sheaths are thinner.

It is believed that early MS disease is driven by inflammation-mediated demyelination and oligodendrocyte damage, as shown in Figure 1, leading to axonal conduction block with limited axonal loss. This CNS damage is thought to be mediated by components of the innate (neutrophils, monocyte-derived-macrophages and natural killer cells) and adaptive (CD4+, CD8+ T cells, and B cells) immune systems. During the relapsing remitting phase of disease, patients undergo sporadic clinical relapses in conjunction with areas of focal CNS neuroinflammation. Patients will normally experience significant recovery from neurologic symptoms during these remissions. Differentiation of oligodendrocyte precursors and subsequent remyelination, as shown in Figure 3A, is thought to underlie these remission periods, particularly in the early stages of MS.

During the progressive phase of the disease, patients cease to experience clinical relapses and instead slowly accrue disability. This is characterized by degenerative changes, including oligodendrocyte and neuronal loss, along with marked tissue atrophy, and a lack of remyelination. Evidence from MRI and histopathological studies now suggests that rather than two independent phases that occur sequentially, both neuroinflammation and neurodegeneration may progress concurrently (8). It is also apparent that we have underestimated the inflammatory component of progressive MS (9). Early in the disease process, advanced MRI shows abnormalities in normal appearing white and gray matter in the absence of focal lesions seen on conventional scans (10). Brain atrophy has been seen in radiographically isolated syndrome, which is the appearance of lesions on MRI without evidence of clinical relapses. Perhaps most convincing is the evidence of brain atrophy at the time of a first clinical attack (11, 12).

For the purposes of this review, we will focus much of our discussion on novel therapies currently in the pipeline for the treatment of the more classically neuroinflammatory condition of MS while remaining cognizant that many of these future therapies are likely to be used in other neuroimmunological indications.

## Novel Therapeutic Targets: Anti-Inflammatories

### Bruton's Tyrosine Kinase Inhibitors

**Figure 2:**
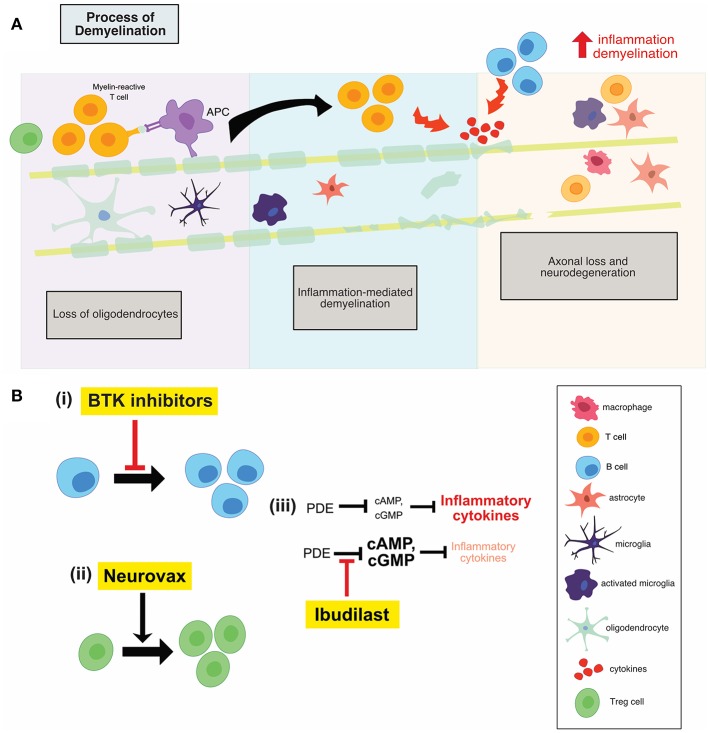
**(A)** Illustration of the process of demyelination in MS which involves the recruitment of adaptive immune cells, the activation of innate CNS immune cells, inflammation mediated demyelination and subsequent neuronal loss. **(B)** An overview of the mechanisms of action of anti-inflammatory therapies. Btk inhibitors (i) reduces B cell proliferation, while neurovax (ii), a trivalent T cell receptor vaccine, stimulates Treg cell activation. Ibudilast (iii) has global anti-inflammatory effects and results in a decrease in the level of circulating inflammatory cytokines. PDE, phosphodiesterase; Btk, Bruton's tyrosine kinase; Treg, regulatory T cells; OPC, oligodendrocyte progenitor cell.

The clinical success of Ocrelizumab, a B cell depleting anti-CD20 monoclonal antibody, has generated a huge amount of interest in further therapeutic targeting of these cells. A more specific, modulatory approach is of particular interest, to target B cell activity in the absence of widespread depletion, thereby alleviating any potential side effect of widespread depletion. Bruton's tyrosine kinase (Btk) is a cytosolic non-receptor tyrosine kinase belonging to the Tec family of kinases (13) and is predominantly expressed by B cells. Btk is critical for B cell development, differentiation, proliferation and survival, as shown in Figure 2B(i). Therefore, Btk-deficient B lymphocytes die prematurely. Following B-cell receptor engagement, Btk translocates to the plasma membrane where it is activated by sequential phosphorylation and auto-phosphorylation (13).

Btk is required for NLRP3 inflammasome activation (14). NLRP3 is a pattern recognition receptor that assembles with its adaptor molecule and catalytic protein (caspase 1) upon activation. Once the assembly is complete, the unit self-cleaves pro-caspase 1 to caspase 1, which then cleaves pro-IL-1B and pro-IL-18 to IL-1B and IL-18 respectively, leading to the induction of pyroptosis (15). Interestingly, expression of caspase 1 and IL-1B is seen in MS lesions and the levels of caspase 1 and IL-18 are increased in MS patients' peripheral blood mononuclear cells (PBMCs) compared to healthy controls. In the mouse model of MS, experimental autoimmune encephalomyelitis (EAE), lack of NLRP3 was shown to be protective. Similarly, IFN-B, a first line therapeutics for MS, functions to suppress NLRP3 inflammasome activation (16, 17). Therefore, inhibition of Btk may be beneficial in MS at least partially through inhibition of the NLRP3 inflammasome.

Btk may also contribute to disease progression through phosphorylation of phospholipase Cy2 and generation of inositol 1,4,5-triphosphate and diacyglycerol. These processes result in downstream calcium influx and subsequent NF-kB activation (18, 19). NF-kB is activated by cytokines TNF and IL-1B, which are also upregulated in MS (20). It is proposed that the NF-kB pathway drives GM-CSF production and secretion by T cells. GM-CSF is crucial for the development of inflammatory demyelinating lesions and for controlling migration and proliferation of leukocytes within the CNS (21). Moreover, GM-CSF may increase production of IL-1B, IL-6, TNF, and nitric oxide (NO) by further upregulating NF-kB (20). Therefore, another mechanism through which Btk inhibitors may exert their beneficial effects is through the inhibition of NF-kB and its downstream signaling pathways.

Mice expressing loss-of-function mutations in Btk (a model of human x-linked immunodeficiency) showed slower induction of EAE and milder disease development than wildtype (WT) mice (22). There was no weight loss reported in these mice after EAE induction, compared with ~13% loss in WT mice. Polymorphonuclear neutrophil granulocytes produced far less NO upon stimulation with LPS compared to LPS-treated WT cells (22). These findings echoed observations from another study in which MOG-induced EAE was suppressed in mice with loss-of-function mutations in Btk (23). Moreover, plaque formation and demyelination were significantly lower compared to WT controls. However, it is important to note that these studies include findings in mice that lacked functional Btk and as such have developmental defects. Therefore, these studies are not highly applicable to humans, since even with Btk inhibitors, some Btk activity remains; EAE studies where Btk remains expressed but is inhibited therapeutically should provide more salience as it pertains to human disease. Several pharmaceutical companies are currently pursuing novel Btk inhibitors with varying levels of brain penetrance, below, we detail the two most advanced compounds, currently in clinical development.

#### PRN2246

PRN2246 is a potent and selective oral Btk inhibitor designed to penetrate the blood brain barrier developed by Principa Biopharma in collaboration with Sanofi. PRN2246 blocks B cell receptor-mediated activation and Fc receptor activation in immune cells and demonstrated dose-dependent protection from disease induction in EAE. Phase I clinical trial evaluating the safety and tolerability of PRN2246 found that the drug, at doses between 7.5 and 120 mg daily, had no serious medication-related adverse events in eighty healthy volunteers (Australian New Zealand Clinical Trials Registry, 2018). PRN2246 was detected in the spinal fluid of the participants, demonstrating its ability to cross the blood-brain barrier. PRN2246 is currently undergoing a phase II clinical trial.

#### Evobrutinib (M2951)

Evobrutinib is a covalent, oral Btk-specific inhibitor (Merck, Germany). In March 2018, promising results from a phase IIb clinical trial were announced, in which it was demonstrated that evobrutinib treatment resulted in a reduction in gadolinium enhancing T1 lesions [indicative of blood brain barrier disruption (BBB)] measured at weeks 12, 16, 20, and 24 in RRMS patients compared to patients receiving placebo (24).

### NeuroVax

One of the main underlying mechanisms contributing to the inflammatory phase of MS is the emergence of pathogenic T cell populations, permitted as a result of reduced immune regulation. This regulation is typically mediated by IL-10-secreting T regulatory 1 cells and natural CD4^+^ CD25^+^ regulatory T cells (Treg) (25). Therefore, development of immune-based vaccines that can restore regulatory mechanisms remains a therapeutic goal. NeuroVax is a trivalent T cell receptor (TCR) peptide vaccine. It is a combination of three TCR peptides (BV5S2, BV6S5, and BV13S1), which appear on the surface of specific myelin-reactive T cells involved in MS pathology (25). This means that the vaccine will elicit an immunological response targeting the harmful, self-reactive T cells. NeuroVax is thought to stimulate FoxP3+ Tregs, as shown in Figure 2B(ii), which have a regulatory function and suppress activity of deleterious autoreactive T cells (25).

In 1989, two independent studies found that vaccination with TCR peptides effectively induced resistance to EAE in rodents (26, 27). Vaccinating Lewis rats with synthetic peptides corresponding to EAE-associated TCR idiotopes resulted in significant resistance to EAE, as none of the animals had clinical or histological signs of the disease, compared to non-vaccinated Lewis rats (26, 27). However, the animals were vaccinated before induction and clinical onset of EAE. This has minimal clinical relevance, since intervention in the human disease starts after disease onset. Importantly, vaccination with TCR peptides in Lewis rats with established EAE reduced clinical severity in these animals from grade 3.2 to grade 1.5 within 48 h and to grade 0.2 within 72 h, compared to non-vaccinated Lewis rats with established EAE (28). Disease duration was also lower in vaccinated rats. These data suggest potential efficacy of vaccination with TCR peptides as a treatment for MS.

Vaccination with TCR peptides, namely NeuroVax, induces a transient increase in a population of IL-10-secreting, TCR-reactive T cells and an increase in FoxP3 expression which is the hallmark of regulatory CD4+ T cell phenotype, as shown in Figure 2B(ii). This was shown in a single-arm study evaluating the effect of 15 injections of NeuroVax over 54 weeks in 27 MS patients (14 RRMS, 10 SPMS, and 3 PPMS) and their age- and sex-matched healthy controls (25). Following three monthly injections, PBMC analysis showed a significant increase in TCR-reactive T cells that produced high levels of IL-10, as well as FoxP3 expression in CD4+ CD25+ T cells returning to healthy control levels, suggesting the drug's capability of boosting specifically reactive T cells that mediate immunosuppression (25) Prior to vaccination, there was a low frequency of proliferating TCR-reactive T cells, which increased significantly after 12 weeks of therapy, and then gradually decreased by the end of the trial. As well, the frequency of IL-10-secreting TCR-reactive cells increased to its maximum after 15 weeks of therapy, and then decreased to starting levels by the end of the 54 weeks (25). It was established that all subjects receiving the tripeptides in incomplete Freund's adjuvant (IFA) had a significant increase in frequency of PBMC T cells reactive to the TCR vaccine, as measured by the proliferation in the limiting dilution assay (25).

NeuroVax is currently undergoing a much larger Phase II clinical trial (NCT02149706), with 150 participants treated with either NeuroVax, consisting of a trivalent TCR peptide formulation in IFA, or IFA alone, via an intramuscular (deltoid) injection once every 4 weeks for 48 weeks. The primary outcome measure is expanded disability status scale (EDSS) in 48 weeks between the two treatment groups. A secondary measurement will evaluate white blood cell count and FoxP3 expression. The trial is randomized, double-blind, and placebo-controlled for subjects with SPMS, results of which are expected to be announced in summer 2019.

### Ibudilast

Ibudilast was first utilized as a treatment for articular rheumatism due to its anti-inflammatory effects (29). Since then, it has been used to treat patients with cerebrovascular disorders based on its inhibitory actions on platelet aggregation. Recent studies have suggested that ibudilast may have neuroprotective properties mediated via an anti-inflammatory mechanism of action and therefore hold potential as a treatment for MS.

Ibudilast is an inhibitor of cyclic nucleotide phosphodiesterases (PDEs) and can readily cross the blood-brain barrier (30). Ibudilast inhibited type I and II PDEs in the extracts from rat brain (30). cAMP activates anti-inflammatory cascades and inhibits T cell immune function (31); PDEs break the diphosphide bonds in cAMP, therefore inactivating the molecule and reducing the activation of anti-inflammatory cascades. As a result, inhibitors of PDEs, such as ibudilast, reduce the inhibition on cAMP, allowing for the activation of anti-inflammatory cascades (31). Treatment with ibudilast resulted in significant suppression of LPS-induced NO and TNF production, both of which are pro-inflammatory mediators produced by microglia and astrocytes (30). Ibudilast also suppressed the proliferation of microglia induced by GM-CSF, M-CSF, and TPA, but it has no effect on unstimulated microglia (30). Therefore, through reducing the inhibition on cAMP, ibudilast may result in lower inflammation and T cell activation which prove beneficial in the MS setting, shown in Figure 2B(iii).

In astrocytes exposed to hydrogen peroxide (H_2_O_2_), ibudilast reduced cytochrome-c release from the mitochondria and inhibited H_2_O_2_-induced increase in caspase-3 activity (32). Ibudilast increased intracellular cyclic GMP (cGMP) levels in astrocytes, when cGMP was inhibited, the protective effects of ibudilast were inhibited (32). Therefore, in addition to reducing the inhibition of cAMP, ibudilast may exert its neuroprotective effects through reducing the inhibition of cGMP.

In rats induced with EAE, the mean clinical score was significantly reduced when treated with ibudilast every day (33). Histopathological scores indicated a significant reduction of inflammatory cellular infiltration in the lumbar spinal cord from ibudilast-treated animals compared to that from control animals. However, when ibudilast was given after the onset of the first clinical sign of EAE, no inhibitory effect was observed (33).

The effects of ibudilast on inflammatory cytokines and Th1 and Th2 cell activation was evaluated in 9 women and 1 man, all of whom had RRMS, in a cross-over study (34). After treatment with ibudilast, there was a significant reduction in the mRNA levels of Th1 cytokines IFN-y and TNF in CD4+ cells. Natural killer T cells (NKT) cell subset significantly increased following treatment, but no change was observed in the placebo and the healthy control groups (34). Indeed, NKT cells in MS are Th2-biased in remission and are markedly reduced in the peripheral blood of the patients during relapse. Therefore, this ibudilast-mediated increase in the NKT subset may underlie ibudilast's clinical effect.

Ibudilast recently underwent a randomized, placebo-controlled phase II trial in progressive MS (35). In this 96-week trial, 108 patients were randomly assigned to receive 100 mg of ibudilast orally, while 112 patients received placebo. Patients on ibudilast had ~2.5 ml less brain-tissue loss as compared to patients receiving placebo. In 2010, ibudilast underwent a multicenter, double-blind, phase II trial, evaluating the efficacy of the drug in 292 patients with RRMS, rather than progressive MS (36). The primary MRI variable of cumulative active lesions over 12 months of treatment did not differ between treatment arms (placebo, 30 mg/d, 60 mg/d). There was no change in the cumulative number of newly active lesions over 24 months between the treatment arms. Percent brain volume change was lower in the treatment groups compared to placebo over 12 months; this effect remained over the 2 years. There was a reduction in the proportion of lesions that evolved into persistent black holes (signifying tissue loss) for both ibudilast groups vs. placebo. In terms of clinical outcomes, ~50% of both ibudilast groups remained relapse-free during the first 12 months, compared to placebo treated. Time to first relapse increased in both treatment groups. Over 2 years, there was less confirmed EDSS progression in those on active treatment throughout the study vs. those initially receiving placebo. Treatment with ibudilast at both concentrations was safe and well-tolerated. Therefore, this study showed that, as evidenced through lower brain volume change and lower progression in EDSS, ibudilast may exert neuroprotective effects.

## Novel Therapeutic Targets: Remyelination/Repair-Promoting

### GNbAC1

Human endogenous retroviruses (HERVs) were integrated into the human genome 30–40 million years ago and are transmitted genetically. Although most HERVs have been rendered inactive through mutations and deletion signals, some are capable of being reactivated to produce viral products (37). Inflammatory stimuli may activate the expression of human endogenous retroviral elements (HERVs) via epigenetic dysregulation (38). For instance, transcription of HERV-W envelope proteins (formerly known as MSRV-env) has been shown to be upregulated in brain lesions of MS patients (39) and there is compelling evidence for an association between HERV expression and MS (40). Indeed, HERV-W env expression can be detected in serum, PBMCs and cerebrospinal fluid (CSF) from MS patients, but not healthy controls (38). Further, HERV-W expression has been shown in astrocytes, microglia and macrophages of MS patients, but not controls (41). It is also associated with areas of active demyelination.

**Figure 3:**
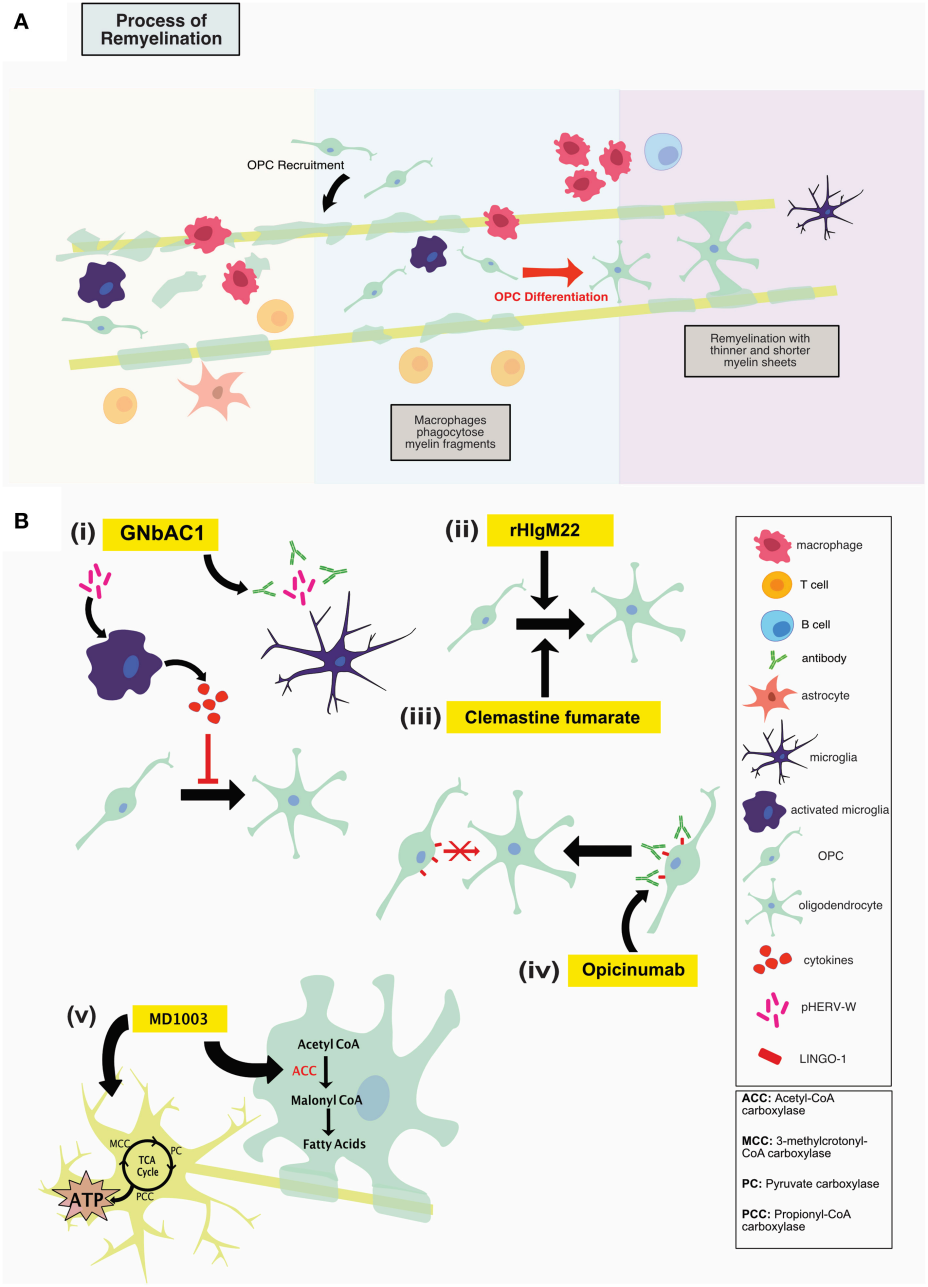
**(A)** Illustration of the process of remyelination in MS which involves the phagocytic clearance of myelin debris by myeloid cells, recruitment of OPCs to the lesion, OPC differentiation and remyelination. **(B)** An overview of mechanisms of action of repair-promoting novel therapeutics. GNbAC1 (i) is a monoclonal antibody, targeting HERV-W, therefore rescuing myelin synthesis. rHIgM22 (ii) is a recombinant human IgM antibody that binds live oligodendrocytes and promotes remyelination by inducing OPC differentiation and proliferation. Clemastine fumarate (iii), a muscarinic receptor antagonist, induces OPC differentiation, and therefore increases remyelination. Opicinumab (iv) is a monoclonal antibody against LINGO-1 (negative regulator of remyelination), and results in enhanced OPC differentiation and remyelination. MD1003 (v), an oral formation of biotin at a high dose, may activate Krebs cycle to increase energy production in demyelinated axons and enhance myelin production in mature oligodendrocytes. Treg, regulatory T cells; OPC, oligodendrocyte progenitor cell.

HERV-W env protein was found to mediate TLR4-dependent induction of proinflammatory cytokine expression in human and murine monocytes (42, 43). Treatment of cultured primary oligodendroglial precursor cells (OPCs) with HERV-W env was shown to result in an overall reduction in OPC differentiation (43). OPCs contribute to the myelin repair process in the adult CNS; blocking their differentiation by HERV-W env protein may therefore disrupt this remyelination. In fact, in MS patients, 88% of HERV+ patients had EDSS score higher than 5 compared to 12% of HERV- patients (44). The HERV-W env mediated inhibition of OPC-directed myelin synthesis could be rescued using a specific HERV-W env-neutralizing humanized immunoglobulin termed GNbAC1, as shown in Figure 3B(i). GNbAC1 is a recombinant humanized monoclonal antibody of the IgG4/kappa class, and it is the only mAb targeting the envelope (env) protein of an active element from HERV-W (45). GNbAC1 can inhibit binding of HERV-W to TLR4, and its downstream activation of inflammation. In fact, treatment of human PBMCs with HERV-W env resulted in a potent and concentration-dependent release of the pro-inflammatory cytokines IL-6 and TNF, an effect that is antagonized by GNbAC1 (46). Treatment with GNbAC1 also antagonized the reduction in CNPase and MBP expression, two mature myelination markers, in human OPCs.

In a phase I, randomized, double-blind, placebo-controlled, dose-escalating clinical trial, GNbAC1 was administered to 33 MS patients (47). The drug was well-tolerated in the patients, and all adverse events were mild or moderate with the adverse events likely to not be drug-related. However, one of the limitations of this study was that the subjects in the study were all Caucasian males. Therefore, the safety of the drug in female patients cannot be commented upon confidently.

In a phase IIa single-blind, placebo-controlled, randomized clinical study, the safety of escalating doses of GNbAC1 was assessed in 10 patients (7 males and 3 female) with RRMS, SPMS or PPMS (48). There was no particular pattern of adverse events that could be attributed to the treatment and there was no difference in the incidence of adverse events between the two dose groups, GNbAC1 was deemed safe for MS patients (48).

### rHIgM22

One approach to promoting remyelination in MS has been immunoglobulin (Ig) therapy, using naturally occurring autoantibodies encoded with germline sequences in the variable region (49). In humans, natural auto-antibodies are typically IgM or IgA (50). Whereas, some natural antibodies bind to neoantigens, others bind to self-proteins and lipids upon structural changes induced by oxidative damage, enzymatic alterations or covalent interactions with altered lipids (49). One type of these natural auto-antibodies, recombinant human IgM22 (rHIgM22) has been shown to bind to oligodendrocytes and actively induce remyelination, as shown in Figure 3B(ii) (51).

In mice treated with cuprizone (a copper chelating reagent which causes oligodendrocyte cell death) the total number of cells of the oligodendrocyte lineage remained the same between controls and those treated with rHIgM22. However, expression of mature oligodendrocyte marker was higher in rHIgM22-treated cuprizone mice and the percentage of OPCs was lower in these mice, compared to untreated cuprizone mice (52). This suggests that the observed effect of rHIgM22 on mature oligodendrocytes is due to enhanced differentiation and not proliferation of OPCs, as shown in Figure 3B(ii). However, *in vitro*, rHIgM22 resulted in an increase in co-localization between oligodendrocyte markers and a proliferation marker in mixed cultures (53). Olig-1 and Olig-2 expression levels were higher in rHIgM22 treated groups, suggesting increased proliferation as a result of rHIgM22. The conflicting findings from these two studies may be due to the different systems used to study the effect of the drug (*in vivo* vs. *in vitro*). rHIgM22 treatment may also result in lower apoptosis by reducing the levels of cleaved caspase 3 and 9, but only in the presence of FGF-2 and PDGF (53). Indeed, treatment of rHIgM22 resulted in lower expression and activity of caspase 3 in spinal cord of TMEV mice (54). Therefore, rHIgM22 treatment may be protective by inducing proliferation and differentiation of OPCs and reducing apoptotic cell death.

rHIgM22, which binds specifically to oligodendrocytes, has been shown to promote remyelination in several animal models of MS (55). Four weeks after treatment of mice with serum HIgM22, virtually all MBP-positive human oligodendrocytes also bound serum HIgM22, demonstrating this antibody's specificity for live oligodendrocytes (51). In the TMEV model of MS, N-acetyl aspartate (found at high concentration throughout the brain) levels and the number of axons and neurons in the brainstem were significantly higher in the group treated with rHIgM22 compared to IgM-treated controls (56). Treatment of rHIgM22 in TMEV mice resulted in a 46% reduction in the lesion load as compared to a 13% increase in the placebo treated group, 5 weeks after treatment (57). Further, in the cuprizone model, the restoration rate of MBP and FluoroMyelin (mature myelin marker) was accelerated in the body of the corpus callosum and the splenium when the animals were treated with rHIgM22, compared to animals treated with cuprizone only (52).

A phase I, multicenter, double blind, randomized, placebo-controlled, dose escalation study was completed in 2017 evaluating the safety and tolerability of single intravenous administrations of rHIgM22 in patients with clinically stable MS (58). Seventy-two patients were enrolled in five dose levels, where the maximum dose was ~15 times the maximally effective dose observed in the TMEV mice. In general, the infusions were well-tolerated among the 55 individuals from the treated group and there were no discontinuations reported. The half-life of the drug increased from 39 to 100 h as the dose increased. Further, rHIgM22 was measurable in the CSF of patients. There were no statistically significant changes in the various clinical or potential pharmacodynamic assessments tested; however, there was a trend toward improvement in patient global impression of change. There was also no change in the CSF analytes of interest, such as MBP, myelin proteolipid protein, and MOG, among others (58). There is currently another phase I clinical trial underway, examining the effect of rHIgM22 on patients with MS immediately following a relapse (clinical trial ID: NCT02398461).

### Muscarinic Receptor Antagonists (Benzatropine, Clemastine Fumarate)

As MS disease course enters a more progressive phase, a failure to remyelinate leads to increased exposure of denuded axons to a chronic inflammatory milieu. Leading to continued neurodegeneration and disability accumulation. Chondroitin sulfate proteoglycan 4+ (NG2), platelet-derived growth factor receptor alpha+ (PDGFR-α) positive OPCs are thought to be the cells responsible for remyelination in the adult brain. These cells migrate to the site of injury and differentiate into mature myelin-forming oligodendrocytes that myelinate denuded axons, providing protection, metabolic support and improving saltatory conduction. While OPCs have the ability to migrate and populate chronic demyelinated MS lesions, these cells appear to experience a differentiation block. Muscarinic receptor antagonists, namely benzatropine and clemastine fumarate, have been shown to induce OPC differentiation, as shown in Figure 3B(iii), and therefore may have therapeutic potential for MS patients.

Benzatropine has been shown to promote OPC differentiation into MBP+ mature oligodendrocytes in a high-content imaging assay (59). Benzatropine is an FDA approved, orally available, anti-cholinergic compound with anti-histamine and dopamine re-uptake inhibition properties. It is believed that benzatropine's ability to induce OPC differentiation is due to antagonism of M1/M3 muscarinic receptors. Benzatropine-induced OPC differentiation was inhibited by the actions of carbachol (a muscarinic receptor agonist). Benzatropine was found to be the most potent of a panel of structurally-diverse muscarinic receptor antagonists with respect to their ability to promote OPC differentiation (59).

In EAE, both prophylactically and therapeutically administered benzatropine decreased the severity of the acute phase of disease and prevented further clinical relapses compared to vehicle. Benzatropine did not seem to affect levels of immune cell infiltration. Areas of immune cell infiltration in benzatropine-treated animals stained positive for myelin, did not show signs of demyelination, and exhibited an increase in mature oligodendrocyte number. Benzatropine induced extensive remyelination compared to vehicle in electron microscopy analysis. Benzatropine's ability to promote remyelination was further evaluated in the cuprizone model of demyelination. Overall, myelin staining (Luxol fast blue staining) and number of mature oligodendrocytes increased significantly in the corpus callosum following benzatropine treatment compared to vehicle-treated animals (59).

Clemastine fumarate, an over the counter anti-histamine and anti-cholinergic molecule, was identified in a novel, high-throughput screening platform to assess OPC differentiation into myelinating oligodendrocytes (60). This screen identified a cluster of 8 FDA-approved antimuscarinic compounds that reduced OPC proliferation while significantly increasing oligodendrocyte differentiation. While benzatropine was also identified in this screen (and clemastine fumarate identified in the previous screen), clemastine fumarate was studied in greater detail due to its ability to cross the BBB and its favorable safety profile. Clemastine's ability to promote remyelination in an *in vivo* setting was examined in the lysolecithin model of demyelination/remyelination. This toxin model produces a well-characterized demyelination event marked by myeloid cell recruitment and activation, astrogliosis, axonal injury, and OPC proliferation and migration. The group reported increased oligodendrocyte differentiation 2 weeks post-lesion. Electron microscopy analysis also revealed faster remyelination kinetics, decreased g-ratios and a 20% reduction in the number of axons left unmyelinated (60).

To better understand the mechanisms behind the beneficial effect of remyelination-promoting anti-cholinergics, this same group set out to uncouple the immunological response (which can also promote remyelination) in EAE from oligodendrocyte-mediated remyelination (61). Prophylactic administration of clemastine in MOG-induced EAE significantly decreased the clinical severity at peak of disease and into the chronic phase. Clemastine treatment caused a significant preservation of both myelin staining and axonal integrity at the later stage of EAE. Clemastine treatment did not alter T-cell/macrophage infiltration or myeloid cell activation, suggesting that the compound's ability to reduce EAE clinical score was not due to an effect on innate or adaptive inflammation. However, an immuno-modulatory effect of the compound beyond affecting cellular migration cannot be ruled out. Muscarinic receptor knockout experiments suggest that clemastine is mediating its pro-differentiation effects through its action on Chrm1. While this data does not preclude the possibility that Chrm1 deletion may modulate an unknown inflammatory role of OPCs, the most likely explanation is that Chrm1 antagonism enhances remyelination, thereby preserving axonal integrity/neuronal function by providing physical and metabolic support (61).

The strong pre-clinical data on clemastine led to the design of a small, single-center, placebo-controlled, crossover study on 50 RRMS patients with chronic demyelinating optic neuropathy (62). Results from this Phase II trial (ReBuild; NCT02040298) were first published in December 2017. Evoked potentials record cortical responses to a repetitive stimulus and can measure the speed of conduction in the CNS, with myelinated axons conducting electrical signals faster than unmyelinated axons. Visual-evoked potentials record cortical responses to a visual stimulus. Most MS patients display demyelination in the visual pathway and thus also a prolongation of visual-evoked potential latency. The primary outcome of the trial (shortening of this latency delay) was met. While reduced latency is only suggestive and does not prove remyelination, these findings are nonetheless very encouraging (62).

Innovative screening techniques have identified anti-cholinergic compounds, of which clemastine fumarate holds the most promise as a class of drug with the potential to enhance remyelination in the chronic demyelinated brain. While the evidence for muscarinic receptors as novel therapeutic targets for the promotion of remyelination is strong, questions do remain. These include concerns around potential side effects of high-dose clemastine, potential for off-target effects, and ultimately its ability to lead to clinical improvement through remyelination in MS patients. These novel molecular targets provide an attractive option given the availability and favorable safety profile of the compounds coupled with the complete lack of efficacious, non-immunomodulatory, myelination-promoting therapies. While optimism is warranted, it is worth noting the observed failure to mobilize OPCs within a hostile cellular milieu (63–65). The ultimate remyelination strategy will likely need to include an anti-inflammatory component to help establish a permissive extracellular environment for OPC mobilization and differentiation. A therapeutic approach that encompasses a concurrent or sequential treatment with both an inflammation targeting molecule and a remyelination promoting therapy would likely hold the most potential for success.

### Anti-LINGO-1

Leucine rich repeat and immunoglobin-like domain-containing protein 1, or LINGO-1, is expressed primarily in the CNS in both oligodendrocyte lineage cells and neurons. Its expression is increased in disease and injurious conditions. LINGO-1 is a negative regulator of the remyelination process through its ability to block differentiation of OPCs into mature myelinating oligodendrocytes, as shown in Figure 3B(iv) (66). This effect seems to be mediated through the RhoA signaling pathway. Antagonizing LINGO-1 as a therapeutic option to boost OPC differentiation has been investigated in several animal models of demyelination. In the cuprizone and LPC toxin models of demyelination treatment with anti-LINGO-1 antibody resulted in a significant increase in myelinated axons as shown by immunohistochemistry and electron microscopy (67, 68). In EAE, LINGO-1 KO mice displayed significantly lower EAE scores as a metric of reduced disease progression and motor abnormalities. These LINGO-1 KO mice also displayed an increased resistance to the development of the disease. In addition, systemically administered anti-LINGO-1 antibody had a positive dose dependant effect on remyelination in a rat model of MOG-EAE (69). In combination these preclinical studies strongly suggest that antagonism of LINGO-1 may hold therapeutic promise in the effort to promote remyelination and repair in the MS brain.

Opicinumab (Biogen Idec; BIIB033) is a first in class, fully human, monoclonal antibody against LINGO-1, as shown in Figure 3B(iv). Efficacy and safety of opicinumab has been examined in two major clinical trials thus far, with a third trial currently underway. Optic neuritis (ON) is inflammatory mediated demyelination in the optic nerve and is often the first clinical manifestation of MS. The primary pathological hallmark of ON is a prolonged visual evoked potential (VEP) latency, caused by demyelination of the optic nerve (70). Opicinumab's ability to enhance remyelination was tested in a randomized, double blind, placebo-controlled, multicenter trial for patients with ON (71). Results from this “RENEW” trial (NCT01721161) showed a trend for latency improvement as compared with placebo. A subgroup analysis did show a significant difference at the later timepoint of 32 in the per protocol treatment group compared with placebo. While there are concerns around the study not being powered to show statistical significance, these results were promising.

The potential efficacy of opicinumab to enhance CNS repair through remyelination and neuronal protection in RRMS and SPMS was assessed in a large randomized, double-blind, study. This “SYNERGY” trial recruited patients already receiving Avonex (interferon beta-1a) and used opiciniumab as an add-on therapy. The primary endpoints of improvement of disability and slowdown of disability progression were not met. This trial did however identify a subgroup of patients that displayed indications of a positive clinical effect. The characteristics that defined this subgroup were a disease duration of >20 years, lower magnetization transfer ratio and diffusion tensor imaging values of the lesions on MRI, indicating higher structural integrity. Based on these findings a new, larger trial is underway launched which focuses on MS patients that fulfill the above criteria (NCT03222973).

### MD1003

MD1003 is an oral formulation of biotin at a high dose (300 mg) of 10,000 times the recommended daily intake (72). Biotin is a member of the B vitamin complex, a water-soluble vitamin that acts as a cofactor for decarboxylase enzymes and can cross the blood brain barrier. High doses of biotin have been a therapeutic option in biotin-responsive basal ganglia disease, an orphan neuro-metabolic disease (73). As well, 5 patients suffering from optic neuropathies and leukoencephalopathy responded clinically to high doses of biotin. Interestingly, one of these 5 patients suffered from SPMS (74). Therefore, these findings warranted a trial of high doses of biotin in patients with progressive MS.

Biotin aids in carbon dioxide transfer to various macromolecules and is associated with several specific enzymes in humans, including pyruvate carboxylase (PC) for liver gluconeogenesis and acetyl-CoA carboxylase for lipid synthesis (72). With respect to MS, MD1003 aims to activate the Krebs cycle to increase energy production in demyelinated axons and enhance myelin synthesis in oligodendrocytes. Biotin is bioavailable, rapidly absorbed and excreted in urine (72). As a cofactor for four essential carboxylases, MD1003 may activate carboxylases to support myelin repair through enhanced synthesis of fatty acids and protect against hypoxia-driven axonal degeneration by enhancing energy production in neurons (75). Indeed, MD1003 is actively transported across the human blood brain barrier via the sodium-dependent multivitamin transporter. Hypothesized mechanisms of action fall under two main categories: promotion of remyelination through enhanced fatty acid synthesis in oligodendrocytes and enhancement of energy production in the CNS, thereby protecting demyelinated axons from cell death, as shown in Figure 3B(v) (75).

#### In Oligodendrocytes

Biotin (MD1003) is a cofactor for ACC1 and ACC2, enzymes involved in fatty acid synthesis. ACC1 catalyzes the rate-limiting step in fatty acid biosynthesis and is primarily expressed in oligodendrocytes. A significant proportion of cytosolic ACC is detectable in purified myelin (75).

Choline, the core component of hydrophilic heads of phospholipids, is commonly elevated in MS plaques and thought to result from the breakdown of the membrane that occurs during characteristic pathogenesis involving inflammation, gliosis and demyelination (75). The choline to creatine ratio was normalized over time with MD1003, a process that is thought to reflect myelin repair accompanied by a decrease in free choline release from membranes. Remodeling of the myelin sheath is thought to occur relatively slowly, which is consistent with the delay in symptom alleviation seen in MS patients treated with MD1003 (75).

#### In the Reversal of Virtual Hypoxia

High-dose biotin may also be targeting cellular energy levels, depletion of which arises from demyelination and is responsible for the progressive and irreversible neuronal degradation in progressive MS. The loss of saltatory conduction increases the energy required for nerve propagation. This shift in propagation mechanisms is accompanied by a need for an increase in mitochondria in the newly demyelinated axons, which is met with a pathological impasse: in MS patients, axonal ATP production is compromised due to neuronal mitochondrial defects (75). This discrepancy between energy requirement and output creates a state of virtual hypoxia, which may act as the trigger for neuron degeneration (75). It is thought that this hypoxic state can be reversed through high-dose biotin in its role as a cofactor for PC, MCC and PCC, enzymes central to aerobic energy production, all of which are known to be expressed in both astrocytes and neurons (75). These biotin-dependent carboxylases feed into the tricarboxylic acid cycle at different entry points; by increasing the available pool of ATP, biotin may act to reduce neural dysfunction and various hypoxic effects, as shown in Figure 3(v) (75).

MD1003 underwent a 12-month randomized, double-blind, placebo-controlled trial followed by an open-label 12-month extension phase with all patients receiving the drug (76). The study included 154 patients with PPMS and SPMS, who received MD1003 (100 mg) or placebo, thrice daily. Thirteen patients out of 103 treated with MD1003 had a reduction in MS-related disability, compared with no patients in the placebo arm. 10/13 patients had improved EDSS score and 5/13 had improved “time to walk 25 feet” times, and 2/13 improved on both scores. As well, 12 of the patients treated with MD1003 had reduced MS-related disability at 18 months and 24 months. 3/42 patients that switched over to MD1003 from placebo also experienced reduced disability at 18 and 24 months. The proportion of patients with EDSS progression at month 9 and 12 was higher in the placebo arm compared to the MD1003 arm. At 12 months, the mean EDSS decreased from baseline in the MD1003 arm but increased at the expected rate in the placebo arm. However, EDSS progression stopped after placebo patients were switched to MD1003. The adverse events that occurred during the study were likely not related to MD1003 treatment. At 12 months, MRI examination identified more new MS-specific lesions in MD1003-treated patients compared to placebo-treated patients, although the difference was not significant (76).

MD1003 is currently in phase 3 clinical trial (NCT02936037), which is a randomized, placebo-controlled, blinded study testing at least 15 months of MD1003 treatment. Hundred milligram biotin capsules will be given to patients thrice daily, and the study is expected to be completed by September 2019. MD1003 is also being tested in separate phase III clinical trial (NCT02220244) to determine potential effects in chronic visual loss related to optic neuritis in MS. Placebo controlled, this study aims to measure the change from baseline of best corrected visual acuity at 100% contrast between treatment groups.

### Edification of Current Therapies

Fingolimod, which binds to the sphingosine-1-phosphate-1 (S1P_1_R) receptor and results in its functional antagonism, was the first approved oral disease-modifying therapy in 2010 (77, 78). Although fingolimod's intended action is through binding of the S1P_1_ receptor, its non-selective modulation of S1P_3_, S1P_4_ and S1P_5_ has led to unwanted adverse effects, resulting in the development of six specific S1P_1_ receptor modulators (77). Mayzent (siponimod) is one such drug with results from its double-blind, randomized phase III SPMS study showing that this once daily oral therapy met its primary endpoint of reduced risk of 3-month confirmed disability progression and reduced 6-month CDP over placebo. By MRI, siponimod slowed the rate of brain volume loss, as well as that of T2 lesion volume over 12 and 24 months (79). Based on these results Mayzant has recently received FDA approval as the first oral therapy to treat active SPMS.

Targeted depletion of CD20+ B cells has also been an effective treatment in MS and several different anti-CD20 monoclonal antibodies have been developed for MS, including rituximab, ocrelizumab, and ofatumumab (80, 81). Indeed, the only FDA-approved treatment for PPMS is ocrelizumab.

Cell-based therapeutic strategies have also been investigated in MS (82), one being immunoablation of autoreactive effector cells, followed by reconstruction of the immune system using autologous hematopoietic stem cell transplantation. A retrospective study indicated that immunoablation followed by hematopoietic stem cell transplantation achieved “no evidence of disease activity” in a greater proportion of MS patients than reported from trials of other available disease modifying therapies (82). Mesenchymal stem cell (MSC) therapy is another form of cell-based therapy, due to evidence showing that MSCs play a significant systemic role in the repair of many tissues. Some studies have reported preliminary evidence of benefit for MS patients, but larger, controlled phase II studies are needed and underway. Glial progenitor cells and induced pluripotent stem cell-induced oligodendrocytes, injected into the CNS, may also offer therapeutic potential through enhancing remyelination in MS. Studies investigating the effect of this form of cell-based therapy are currently underway (82).

## Discussion

Neuroimmunological diseases, such as MS, myasthenia gravis, Guillain-Barré, and NMO are all associated with classical neuroinflammation. Up until recently, preventing damage in the CNS by regulating peripheral immunity has been the central method of treatment. A current focus of the field is the development of novel methods to promote repair and remyelination. In this review, we have discussed three novel immunomodulatory therapeutics and five novel therapies that promote repair. A summary of all drugs is presented in Table 1.

**Table 1:** Summary of three novel immunomodulatory therapeutics and five novel therapies that promote repair in MS.

**Drug**	**Classification**	**Target/mechanism of action**	**Results from animal model studies**	**Results from clinical trials**
**NOVEL THERAPEUTIC TARGETS: ANTI-INFLAMMATORIES**				
PRN2246/M2951	Bruton tyrosine kinase (Btk) inhibitor	Inhibition of BTK→ inhibition of inflammasome→ ↓IL-1B and IL-18 Inhibition of BTK→ ↓NF-kB→ ↓inflammation	Genetic ablation of Btk: ↓Weight loss, ↓NO production, ↓EAE, ↓clinical score	PRN2246—Phase 1 clinical trial - 7.5−120 mg daily - 80 healthy volunteers - No serious medication-related adverse events - Detected in the spinal fluid of participants M2951 phase 11b clinical trial - Reduced gadolinium enhancing T1 lesions in RRMS patients
Neurovax	Trivalent TCR peptide vaccine	Stimulates FoxP3 positive T cells (regulatory T effector cells)→ ↓autoreactive T cells	Induced resistance to EAE when treatment starts before or after clinical onset of EAE	Phase I/II clinical trial - 100% of the patients responded to NeuroVax - Disease activity was not different after vaccination
Ibuldilast	Phosphodiesterase inhibitor	Inhibits PDE→ ↑cAMP→ ↓inflammation, ↓T-cell immune activation, ↓microglia activationInhibits PDE→ ↑cGMP→ ↓ H_2_O_2_-induced astrocytes	↓Clinical score in EAE mice ↓Inflammatory cellular infiltration in the spinal cord	Phase II in progressive MS - 108 patients - 100 mg of ibudilast each day - Patients on ibudilast had 48% less atrophy progression of their brain tissue Phase II in RRMS - 292 patients - 30 mg/d or 60mg/d - ↓ volume change in the brain in those treated with ibudilast - ↓proportion of lesions evaluated into persistent black holes - ↑time to first relapse - ↓increase in EDSS
**NOVEL THERAPEUTIC TARGETS: REPAIR PROMOTING**				
GNbAC1	Humanized neutralizing IgG4 antibody	Promotes restoration of protective myelin coating by blocking pHERV-W	↑Lifespan after MOG treatment in mice ↓Clinical score	Phase I clinical trial - 6 different doses - 33 MS patients - Drug was well tolerated - Only white males Phase IIa clinical trial - 10 patients (male and female) - RRMS, SPMS and PPMS - Safe for patients
rHIgM22	Serum form of human IgM 22	Monoclonal antibody binds→ only to oligodendrocytes ↑OPC differentiation and proliferation ↑Calcium influx into the cells	TMEV model; ↑Remyelination ↓Lesion load Cuprizone model ↑Restoration rate of MBP	Phase I clinical trial - 72 patients - Clinically stable MS - Infusions were well-tolerated - Crossed BBB
Clemastine fumarate	Antihistamine	Remyelination, immunomodulator; promotes development of oligodendrocytes	↓Clinical severity of EAE ↑Preservation of myelin	Phase II clinical trial - 50 patients with RRMS - Safe for patients - Increased feelings of fatigue
Opicinumab	Anti-LINGO-1	Enhances remyelination by inhibiting LINGO-1, a negative regulator of OPC differentiation	↑Myelinated axons in cuprizone model and MOG-EAE rats ↓EAE scores and disease progress	Phase II clinical trial - RRMS and SPMS patients - Primary endpoints not met - Subgroup (>20 y disease duration, lower MTR and DTI) showed positive clinical effect
MD1003	Highly concentrated form of biotin	Activates Krebs cycle→ ↑energy production ↑Mxyelin repair May reverse hypoxia	N/A	Clinical trial: - 154 patients with SPMS and PPMS - 100 mg pills thrice daily - ↓In MS-related disability in 13 patients - ↓EDSS progression in treated group

Btk inhibitors, Neurovax, and ibudilast are three novel therapeutics currently in clinical trial, with anti-inflammatory and “damage prevention” modalities. Btk inhibitors, such as PRN2246 and evobrutinib, reduce inflammation by inhibiting activation of NLRP3 inflammasomes or NF-kB, both of which are implicated in MS pathology. Both drugs are currently undergoing clinical trials. NeuroVax, is a combination of three TCR peptides, and exerts its neuromodulatory effects by stimulating Tregs and suppressing activity of deleterious autoreactive T cells. Ibudilast exerts its anti-inflammatory and neuroprotective effects via inhibition of phosphodiesterases, thereby allowing cAMP to initiate anti-inflammatory cascades.

GNbAC1, rHIgM22, clemastine fumarate, Anti-LINGO-1 (opicinumab), and MD1003 are the five novel therapeutics currently in clinical trial, which promote repair and show a capacity to induce remyelination. GNbAC1 is a humanized IgG4 antibody that inhibits HERV-W, which is implicated in MS and can lead to OPC apoptosis and remyelination failure. By inhibiting HERV-W, GNbAC1 treatment may lead to higher rates of remyelination. rHIgM22 is a human monoclonal IgM that binds to the surface of live oligodendrocytes and promotes remyelination. Through antagonizing muscarinic receptors, clemastine fumarate induces OPC differentiation. LINGO-1 is a negative regulator of the remyelination process and treatment with opicinumab resulted in increased remyelination. Finally, MD1003 is an oral formation of high dose biotin which may activate the Krebs cycle to increase energy production in demyelinated axons and enhance remyelination.

## Limitations and Future Directions

Some clinical trials investigating the novel therapeutics discussed in this review predominantly recruited Caucasian males. Due to inherent biological differences between males and females, the findings of these studies cannot be accurately and confidently extrapolated to the female population. Further, especially in the case of phase I studies, the number of participants is limited due to the nature of the study, therefore begetting a type II error where the effect of the drug may have been missed.

The exact biological mechanisms of action underlying the drugs' respective functionalities have yet to be demonstrated. Therefore, well-designed mechanistic studies in well-validated animal models and primary human cells are needed. Finally, there is currently a push to repurpose approved, safe compounds that have been used in other indications. Such compounds include amiloride, fluoxetine, and riluzole (83).

## Conclusion

With the emergence of highly effective anti-B cell therapies, it has now become possible to significantly limit the formation of new lesions in RRMS patients and a subset of younger progressive MS patients. The complex relationship between neuroinflammation and neurodegeneration remains poorly understood. However, as current patient cohorts spend longer on the newer generation of immunomodulatory compounds, we will begin to understand if preventing inflammatory activity early in RRMS ultimately delays and/or prevents progression. The clinical experience with these newer compounds will also reveal potential safety concerns that may not be evident over the short or medium timespan. As it stands, it is likely that the next breakthrough in the treatment of neuroimmunological disease will be a biologic with remyelinating or repair-promoting effects. Much of the focus has been directed toward strategies to promote the differentiation of OPCs to myelinating oligodendrocytes; however, recent evidence points to the potential for surviving mature oligodendrocytes to re-wrap denuded axons (84). An observed lack of integration of new oligodendrocytes into so-called remyelinating “shadow plaques” downplays a role for OPC differentiation as the primary cellular event driving remyelination in the MS brain (85). Efforts to induce remyelination from surviving but quiescent oligodendrocytes may be achieved in a cell autonomous manner or in a cell non-autonomous manner by altering the inflammatory microenvironment of a lesion to permit such remyelination. Combatting the multifactorial pathophysiology of progressive MS remains a significant challenge in the field. Success in this area will likely require a holistic therapeutic approach with a compound that incorporates both anti-inflammatory and remyelinating functions.

## Author Contributions

LH conceived the idea and supervised the process. MK, NF, NK, and LH wrote the manuscript. NF generated the figures. All authors revised and proof-read the manuscript.

### Conflict of Interest Statement

The authors declare that the research was conducted in the absence of any commercial or financial relationships that could be construed as a potential conflict of interest.

## Abbreviations

Btk: Bruton's tyrosine kinase
EAE: experimental autoimmune/allergic encephalomyelitis
EDSS: expanded disability status scale
HERV: human endogenous retroviral elements
IFA: incomplete Freund's adjuvant
MBP: myelin basic protein
MOG: myelin oligodendrocyte glycoprotein
MS: multiple sclerosis
NMO: neuromyelitis optica
NO: nitric oxide
OPC: oligodendrocyte progenitor cells
PBMC: peripheral blood mononuclear cells
PBVC: percent brain volume change
PDE: phosphodiesterases
PLP: proteolipid protein
PPMS: primary progressive multiple sclerosis
RRMS: relapsing-remitting multiple sclerosis
SPMS: secondary progressive multiple sclerosis
TCR: T cell receptor
TMEV: Theiler's murine encephalomyelitis virus
Treg: regulatory T cells.
